# Characterization of retinal biomechanical properties using Brillouin microscopy

**DOI:** 10.1117/1.JBO.25.9.090502

**Published:** 2020-09-26

**Authors:** Yogeshwari S. Ambekar, Manmohan Singh, Giuliano Scarcelli, Elda M. Rueda, Benjamin M. Hall, Ross A. Poché, Kirill V. Larin

**Affiliations:** a University of Houston, Department of Biomedical Engineering, Houston, Texas, United States; b University of Maryland, Fischell Department of Bioengineering, College Park, Maryland, United States; c Baylor College of Medicine, Department of Molecular Physiology and Biophysics, Houston, Texas, United States

**Keywords:** retina, Brillouin microscopy, tissue biomechanics, *N*-methyl-D-aspartate, retinal damage, retinal ganglion cells

## Abstract

**Significance:** The retina is critical for vision, and several diseases may alter its biomechanical properties. However, assessing the biomechanical properties of the retina nondestructively is a challenge due to its fragile nature and location within the eye globe. Advancements in Brillouin spectroscopy have provided the means for nondestructive investigations of retina biomechanical properties.

**Aim:** We assessed the biomechanical properties of mouse retinas using Brillouin microscopy noninvasively and showed the potential of Brillouin microscopy to differentiate the type and layers of retinas based on stiffness.

**Approach:** We used Brillouin microscopy to quantify stiffness of fresh and paraformaldehyde (PFA)-fixed retinas. As further proof-of-concept, we demonstrated a change in the stiffness of a retina with N-methyl-D-aspartate (NMDA)-induced damage, compared to an undamaged sample.

**Results:** We found that the retina layers with higher cell body density had higher Brillouin modulus compared to less cell-dense layers. We have also demonstrated that PFA-fixed retina samples were stiffer compared with fresh samples. Further, NMDA-induced neurotoxicity leads to retinal ganglion cell (RGC) death and reactive gliosis, increasing the stiffness of the RGC layer.

**Conclusion:** Brillouin microscopy can be used to characterize the stiffness distribution of the layers of the retina and can be used to differentiate tissue at different conditions based on biomechanical properties.

## 1 Introduction

The neural retina is the thin, light-sensitive innermost layer of the eye and is essential for image formation. The retina is a part of the central nervous system and is composed of a single glial population as well as six neuronal cell types. Among these cells, retinal ganglion cells (RGCs) convey visual information, which is transmitted through the optic nerve to the visual cortex. Retinal diseases, such as glaucoma and retinal ischemia, are significant causes of blindness worldwide. In these conditions, RGCs die and vision is permanently lost since they cannot be replaced.^1^ Thus, to inform new and effective treatments for such diseases, there is a tremendous need to better understand the early pathophysiology leading to RGC cell death. Additionally, the development of noninvasive, early diagnostics would be tremendously beneficial for guiding clinical intervention before permanent RGC loss.

The retina constantly experiences static and dynamic forces^2^ because it is in contact with the vitreous humor, eye movements exert forces leading to stresses on the retina,^3^ and constant pumping of fluid from the retinal pigment epithelium to the choroid exerts negative pressure on the outer wall of the retina.^4^ This prolonged stress can damage the vasculature and neurons,^5^ which in turn can lead to blindness.^6^ Specifically, Müller glial cells are mechanosensitive^7^ and may be especially susceptible to mechanical damage,^8^ which may further exacerbate retinal damage via inflammatory signal cascades and loss of normal retinal homeostasis.^9^

Cell replacement therapies for retinal diseases have been developed and are underway in various stages of clinical trials.^10^ It is well known that the biomechanical properties of the surrounding environment have a profound effect on cellular physiology,^11^ including stem cell growth and the integration of new retinal neurons.^12^^,^^13^ Therefore, biomechanical characterization of the retina could improve disease detection and provide insight into disease etiology and guide new therapeutic interventions.

Compared with the choroid and sclera, the retina is softer and much more fragile, so assessing its biomechanical properties is a challenge. Early studies on retinal elasticity utilized conventional mechanical testing,^14^ but mechanical testing is generally destructive, which often results in tearing of the retina. Atomic force microscopy (AFM) has been utilized to characterize the biomechanical properties of the retina.^15^ For example, AFM showed that among other factors, cellular density is a source of mechanical heterogeneity in the retina^16^ and that the inner limiting membrane stiffness increases during embryonic development in chicks and mice.^17^ Although AFM is capable of nanoscale characterization of tissue stiffness, it requires contact and has lengthy imaging times. Thus, various noninvasive techniques for characterizing retinal biomechanical properties have been proposed. Traditional elastography techniques, such as magnetic resonance elastography^18^ and ultrasound elastography,^19^ generally lack the resolution for precise characterization of the retina, particularly the layers of the retina. Optical coherence elastography (OCE)^20^ has shown promise for characterizing the biomechanical properties of the retinal layers, but it is semiquantitative and mechanical excitation safety concerns need to be addressed.^21^

Brillouin microscopy is a noninvasive, high-resolution, optical elastography technique that has shown promising applications in determining tissue elasticity.^22^ Brillouin microscopy has the benefits of requiring no external excitation and, thus, is an all-optical elastography technique that is capable of subcellular resolution^23^ at safe energy levels for cells and tissues.^24^

This study demonstrates the layer-by-layer biomechanical characterization of retinal tissue stiffness. Brillouin microscopy was used to measure the change in the Brillouin modulus between fresh and paraformaldehyde (PFA)-fixed retinal explants. We also performed biomechanical characterization of a mouse retina with N-methyl-D-aspartate (NMDA)-induced damage. By mimicking the action of the neurotransmitter glutamate and promoting overactivation of the NMDA receptor, intraocular injection of NMDA results in excitotoxicity leading to RGC death.^25^ In response to this damage, Müller cells and astrocytes within the retina activate a protective event called reactive gliosis, where they attempt to safeguard the retinal structure and immune privilege. As part of this process, these cells upregulate the production of intermediate filament proteins, such as glial fibrillary acidic protein and vimentin, and become rigid, forming a glial scar.^26^ Here, we demonstrate that Brillouin microscopy can detect local changes in retinal stiffness that coincide with RGC loss and reactive gliosis.

## 2 Materials and Methods

### 1.1 Sample Preparation

**Fig. 1:**
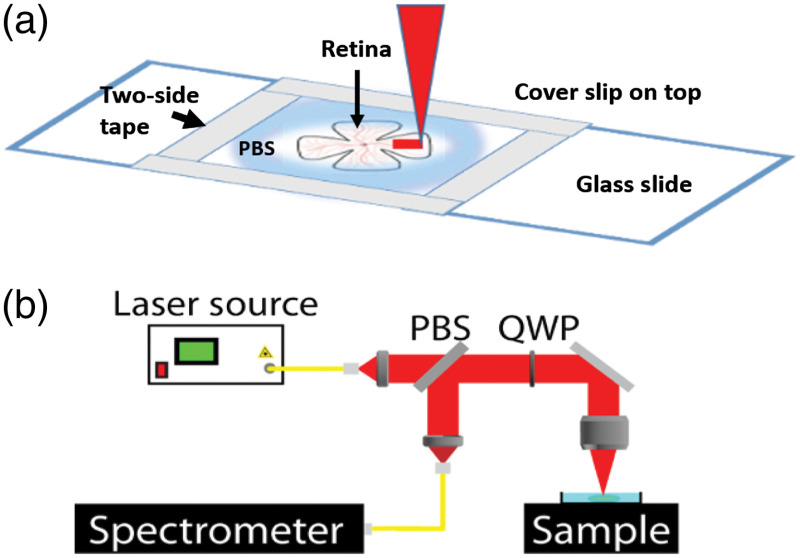
(a) Retinal flat-mount preparation and imaging paradigm. (b) Schematic of the Brillouin microscopy system. PBS, polarization beam splitter; QWP, quarter-wave plate; and spectrometer, two-stage VIPA spectrometer.

All procedures were approved by the Baylor College of Medicine Institutional Animal Care and Use Committee. Adult C57/BL6J mice were euthanized by ${\mathrm{CO}}_{2}$ inhalation and decapitation. Eyeballs were enucleated and kept in 0.1 M phosphate buffered saline (PBS) on ice. Retinas were dissected out and separated into experimental groups (N=2 fixed and N=3 fresh). For fixation, the samples were immersed in 4% PFA at 4°C for 1 h and then washed with 1× PBS. Once the samples were fixed, they were transferred to a 4°C 1× PBS bath. The retinas were mounted flat on a microscope slide, as shown in Fig. 1(a). A well was created on the slide with double-sided tape to maintain sample hydration using 0.1 M PBS at room temperature throughout the experiment. The experiments were performed within 6 h postdissection. Before Brillouin imaging, the thickness of the retina was first determined by an OCT system.

Four-week-old C57/BL6J mice were injected intravitreally with 2 *μ*L of 100 mM NMDA. The mouse was euthanized 48 h postinjection, and the eye-globes were enucleated. Then, the retinas were dissected and mounted fresh on a glass slide as described earlier.

### 2.2 Brillouin Microscopy

Imaging was performed with a home-built Brillouin microscopy system based on a two-stage virtually imaged phased array (VIPA) spectrometer.^27^ The Brillouin system utilized a single-mode 660-nm laser (Torus, Laser Quantum Inc., Fremont, CA, USA) with incident power on the sample of ∼11  mW. The camera exposure time was 0.1 or 0.2 s during measurements depending on the signal quality. The Brillouin microscope utilized a 20× microscopic objective with a 0.42 numerical aperture to focus the laser beam onto the sample. The lateral resolution was ∼1.8  μm and the axial resolution, as defined by Rayleigh range, was ∼10  μm as measured using a beam viewer (LaserCam-HR II, Coherent Inc., CA, USA). Standard materials (water, acetone, and methanol) were used for system calibration before every measurement. The backscattered light from the sample was sent into the VIPA-based spectrometer and detected with an electron-multiplying charged coupled device camera (iXon Andor, Belfast, UK). The sample was scanned in two dimensions using a motorized 3D stage. The Brillouin modulus was mapped along the axial direction of the retinas over ∼300  μm with 2  μm steps and laterally over ∼100  μm also with 2  μm steps. The Brillouin frequency shift observed at each location within the mouse retina was converted to longitudinal elastic modulus, called Brillouin modulus, M, by^28^

$$M=\frac{\rho \lambda^{2}\omega^{2}}{4\eta^{2}\;\sin^{2}(\frac{\theta }{2})},$$ (1)

where ρ is the density of the sample ($\rho =1.033\;g/{\mathrm{cm}}^{3}$ for retina), η is the refractive index (η=1.33),^29^ λ=660  nm is the wavelength of the laser source, ω is the detected Brillouin frequency shift, and θ=180  deg is the light scattering angle. Although the retina does have heterogeneous refractive indices and densities, we assumed a uniform refractive index and density for simplification. Because the refractive index and density are linearly related in tissues, the small variations in each of these do not significantly affect the Brillouin modulus estimation even in the presence of large individual variations as shown in ocular tissues^30^ and by varying the osmotic pressure of cells.^23^ Segmentation of retinal layers was performed manually.

## 3 Results

**Fig. 2:**
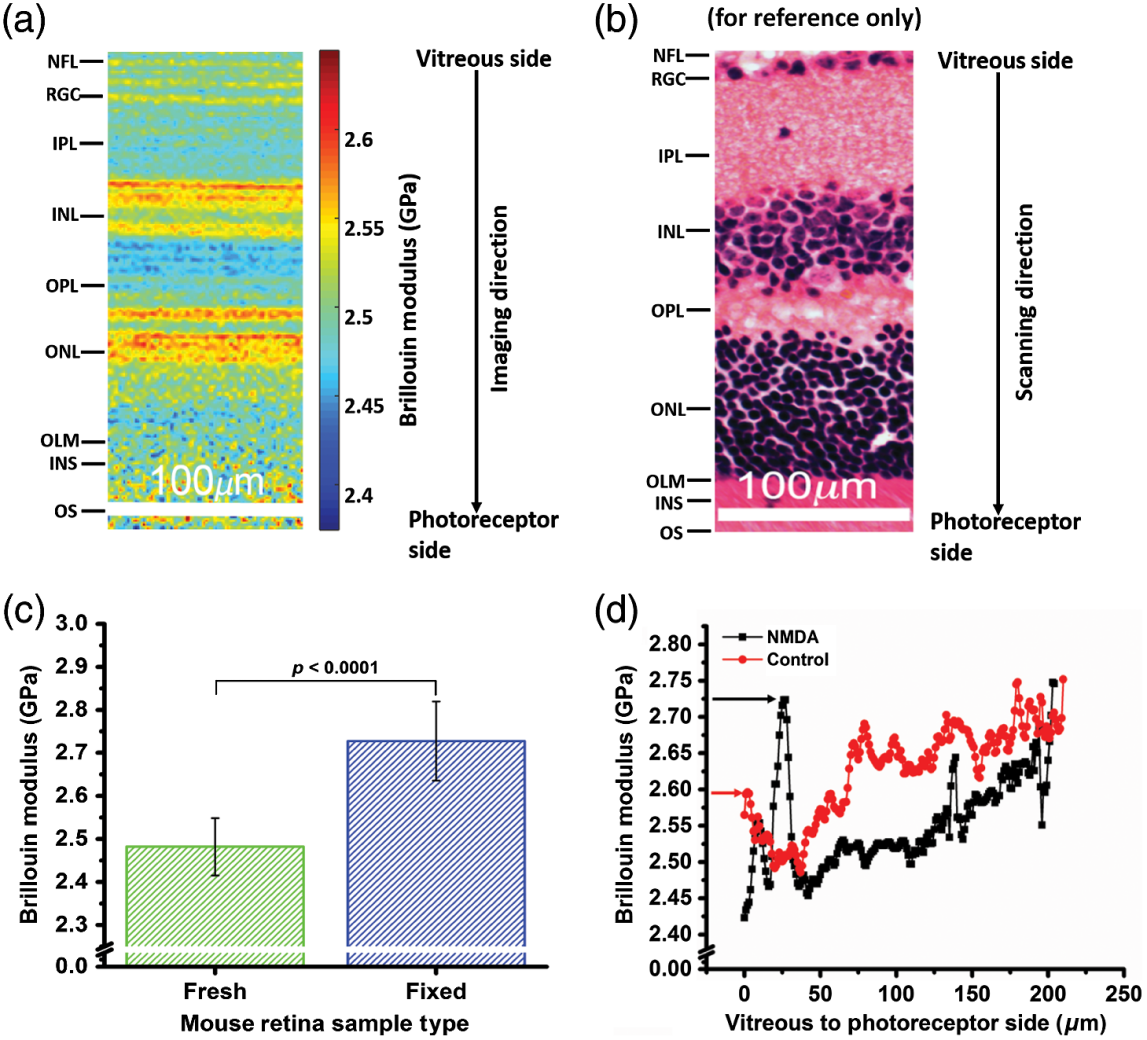
(a) Brillouin frequency-shift map of the mouse retina, (b) histology of mouse retina (for reference only), (c) average Brillouin modulus of fresh (n=3) and fixed (n=2) mouse retina samples (averaged pointwise per sample and then sample-wise per type of sample), and (d) Brillouin modulus depth profile of an NMDA-induced damaged retina and its contralateral control. Arrow indicates the RGC layer.

Figures 2(a) and 2(b) show a section of a typical Brillouin frequency map for a fresh mouse retina alongside a typical histological image of a mouse retina with the different layers: nerve fiber layer (NFL), RGC, inner plexiform layer (IPL), inner nuclear layer (INL), outer plexiform layer (OPL), outer nuclear layer (ONL), outer limiting membrane, photoreceptor inner segment, and photoreceptor outer segment. OCT imaging showed that the thickness of the retina was ∼250 to ∼300  μm. The longitudinal Brillouin moduli of NFL/RGC (n=1950, where n is the number of points in Brillouin scan), IPL (n=1800), INL (n=1900), OPL (n=1650), and ONL (n=2100) were 2.51±0.02  GPa, 2.49±0.01  GPa, 2.53±0.02  GPa, 2.48±0.02  GPa, and 2.53±0.02  GPa, respectively, for the fresh retina sample shown in Fig. 2(a). Next, we compared the longitudinal Brillouin modulus of fresh (N=3) and fixed (N=2) murine retina samples. The Brillouin modulus was first averaged pointwise for each sample and then averaged for each sample type. The results in Fig. 2(c) show that the fixed mouse retinas were significantly stiffer compared with the fresh samples. The average Brillouin modulus over the entire depth of all of the fixed samples was 2.73±0.09  GPa, and for the fresh samples, it was was 2.48±0.06  GPa. A t-test showed that the Brillouin modulus was significantly different (p<0.0001).

To demonstrate a proof-of-concept for detecting retinal damage with Brillouin microscopy, we characterized the biomechanical properties of NMDA-induced damage to the mouse retina. The dissected retinas were kept on a glass slide where the top layer was the NFL/RGC, and the photoreceptor side was at the bottom. Figure 2(d) shows that the NMDA-damaged retina had a very high Brillouin modulus at the RGC layer as compared with the contralateral control retina sample. A greater Brillouin modulus can be the characteristics of dead cells as they are stiffer than live cells,^31^ so the prominent spike in the RGC layer may indicate the loss of cells in this layer by NMDA-induced toxicity. The stiffness change may also reflect reactive astrocytes within the NFL.^32^

## 4 Discussion

In this work, we demonstrated all-optical noninvasive elastography of fresh murine retinal tissue using Brillouin microscopy. The retina is a layered heterogeneous tissue, and each layer has different characteristic biomechanical properties, which are demonstrated by our results. The stiffness of the layers is highly dependent on cell body density.^15^^,^^16^ For example, the nuclear layers have the highest cell body density, so they were stiffer compared with the plexiform layers, which are primarily composed of axons and dendrites. Previous work has shown that the nucleus has a greater Brillouin modulus compared with the other parts of the cell, so it can be inferred that more nuclei would increase the overall stiffness of the corresponding layer.^23^

In the second part of this work, we compared the biomechanical properties of PFA-fixed and fresh mouse retinas. PFA is a cross-linking fixative that covalently bonds proteins within tissue, thereby conferring rigidity.^33^ As expected, the PFA-fixed samples showed a higher overall Brillouin modulus compared with the fresh samples.^34^ However, changes in the hydration state can be a confounding variable in the Brillouin modulus.^35^^,^^36^ Although the VIPA-based spectrometer is sensitive enough to remove the effects of hydration,^37^ validation by another technique, such as AFM, will be the next step of our work.

Finally, we performed a pilot study on the effects of NMDA-induced retinal damage on the stiffness of the retina in a mouse model. The tissue was assessed 48-h post-NMDA injection in a young mouse. The results showed a very drastic increase in the Brillouin modulus of the RGC layer and/or NFL in the damaged retina as compared with its contralateral control. NMDA can induce damage to retinal blood vessels,^38^ but histology, immunostaining, electron microscopy, and OCT have shown that NMDA also results in RGC death and a consequential increase in reactive gliosis.^25^^,^^39^ Our results showed an increase in the Brillouin modulus in only the RGC layer, whereas retinal blood vessels are present in many layers, indicating that the change in stiffness that we detected was most likely NMDA-induced RGC death and possibly the gliotic response. We also observed an increase in the INL Brillouin modulus [∼130  μm in Fig. 2(d)]. While the majority of research has been focused on NMDA-induced RGC death, our results show that the INL may also be damaged and will be an avenue of our future research.

OCT is used routinely for live imaging of retinal damage, but biomechanical assessments of retinal tissue are generally qualitative only, rely heavily on other factors such as geometry,^21^ and have mechanical resolutions a few orders of magnitude greater than the optical resolution. Other high-resolution imaging modalities, such as histology, immunostaining, and electron microscopy, are not suitable for live imaging or the biomechanical assessment of the retina. Although a relatively high power was used for Brillouin imaging, confocal imaging analysis showed no difference in Brillouin imaged and unimaged retinas, and previous research has shown no damage to cells until a much higher incident power.^40^

One major limitation of this work is that Brillouin microscopy can only provide measurements of the tissue bulk modulus instead of the quantitative measurement of elasticity. We have previously demonstrated quantitative measurements of crystalline lens elasticity by combining Brillouin microscopy with OCE.^41^ Performing such measurements on the retina is the next step of our work for truly quantitative measurements of retinal elasticity as well as co-registered structural and biomechanical maps of the retina.

## 5 Conclusion

We noninvasively characterized retinal tissue stiffness using Brillouin microscopy on freshly harvested mouse retinas. The results show that Brillouin microscopy can map the mechanical properties of the different retinal layers. The stiffness of the layers correlated with the cellular density, and fixing the retinas in PFA also increased the stiffness. Furthermore, we demonstrated an increase in the stiffness of the RGC and/or NFL layer due to NMDA-induced damage in a mouse model. This work was a preliminary study to demonstrate noninvasive biomechanical characterization of retinal tissue using Brillouin microscopy. Our future work is focused on *in vivo* studies of the long-term effects of NMDA-induced retinal damage in a mouse model and combining Brillouin microscopy with OCT for noninvasive and all-optical co-registered structural and biomechanical maps of the retina.
